# Susceptibility Weighted Imaging (SWI) Recommended as a Regular Magnetic Resonance Diagnosis for Vascular Dementia to Identify Independent Idiopathic Normal Pressure Hydrocephalus Before Ventriculo-Peritoneal (V-P) Shunt Treatment: A Case Study

**DOI:** 10.3389/fneur.2019.00262

**Published:** 2019-03-29

**Authors:** Wen-Qing Huang, Hui-Nuan Lin, Qing Lin, Chi-Meng Tzeng

**Affiliations:** ^1^Translational Medicine Research Center, School of Pharmaceutical Sciences, Xiamen University, Xiamen, China; ^2^Department of Neurology and Center for Brain Research, The First Affiliated Hospital of Xiamen University, Xiamen, China; ^3^School of Medicine, Xiamen University, Xiamen, China; ^4^The First Clinical College of Fujian Medical University Fuzhou, China; ^5^INNOVA Cell: TDx/Clinics and TRANSLA Health Group Yangzhou, China

**Keywords:** susceptibility weighted imaging (SWI), idiopathic normal pressure hydrocephalus (INPH), vascular dementia (VD), leukoaraiosis (LA), cerebral amyloid angiopathy (CAA)

## Abstract

Idiopathic normal pressure hydrocephalus (INPH) with comorbid vascular dementia (VD) often have poor response to ventriculo-peritoneal (V-P) shunt. Here, three patients over the age of 60 came to the hospital with the similar clinical symptoms, Evan index over 0.3, mini-mental state examination (MMSE) score <27, and cerebrospinal fluid (CSF) pressure under 200 mmH_2_O. They accepted conventional brain imaging scanning, followed by magnetic resonance-susceptibility weighted imaging (MR-SWI) scanning. We found that MR-SWI could distinguish INPH from leukoaraiosis (LA) and cerebral amyloid angiopathy (CAA), through cerebral microbleed (CMB) images, sharply. We highly recommended incorporation of MR-SWI into INPH international guidance as a routine pre-operative diagnostic method preceding V-P shunt treatment.

## Introduction

Idiopathic normal pressure hydrocephalus (INPH) was first reported by Hakim and Adam in 1965 (1). It is a sympathetic treatable and reversible dementia by ventriculo-peritoneal (V-P) shunt (2, 3). The current diagnosis of INPH is primarily based on clinical symptoms and brain fluid-attenuated inversion recovery (FLAIR)-magnetic resonance (MR) imaging. To distinguish INPH from other neurodegenerative diseases and vascular dementia (VD), in adults over 60, had been a considerable challenge. That is why many INPH patients have a poor response to V-P shunt surgery. Leukoaraiosis (LA) and cerebral amyloid angiopathy (CAA) are fairly specific to older people and often co-exist with INPH. Since they share the same clinical symptoms and MR imaging, accurate diagnosis of INPH has become essential to prediction of patient reaction and monitoring patient responsiveness to V-P shunt operations.

The development of functional magnetic resonance imaging (fMRI) makes it possible for us to concretely identify VDs. As SWI has become practical, cerebral microbleeds (CMBs), defined as hemorrhagic microvascular lesions or microangiopathy in the brain, are commonly observed in patients with LA and CAA, respectively (4–6). Here, we demonstrated three typical cases who received a standard scanning including T1-weighted MR, T2-weighted MR, and FLAIR-MR imaging to show the similarities and difference of the MRI and VD symptoms among INPH, LA, and CAA, to provide the best clinical reference for neurosurgical decisions.

## Case Presentation

### Case 1

**Figure 1:**
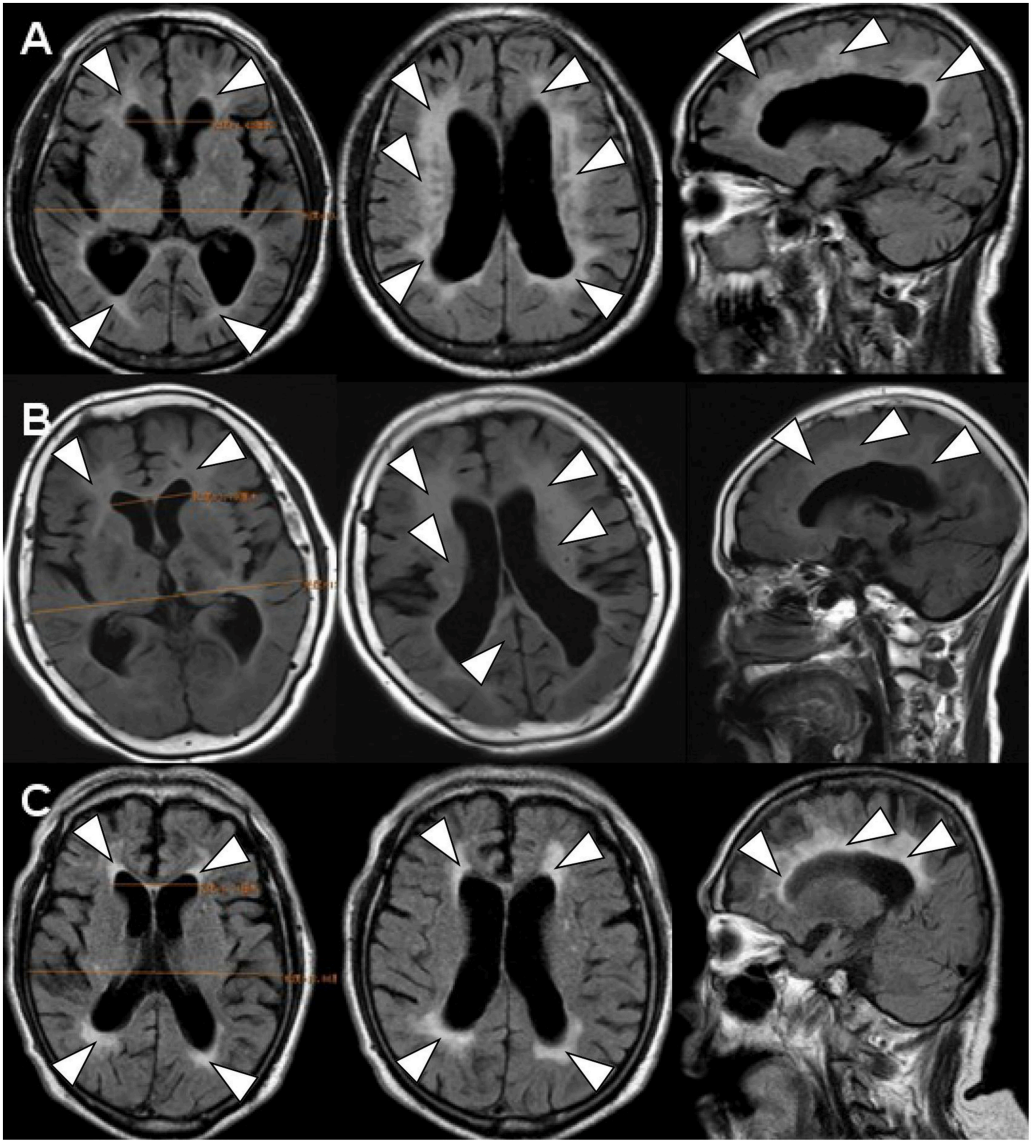
The brain FLAIR-MR imaging. FLAIR-MRI demonstrated the similar image feature of ventricular dilation (Evan index >0.3) (1) and periventricular (2) and deep white matter changes (3) in all of three cases. **(A)** denoted as INPH (case 1); **(B)** denoted as LA (case 2); **(C)** denoted as CAA (case 3).

**Figure 2:**
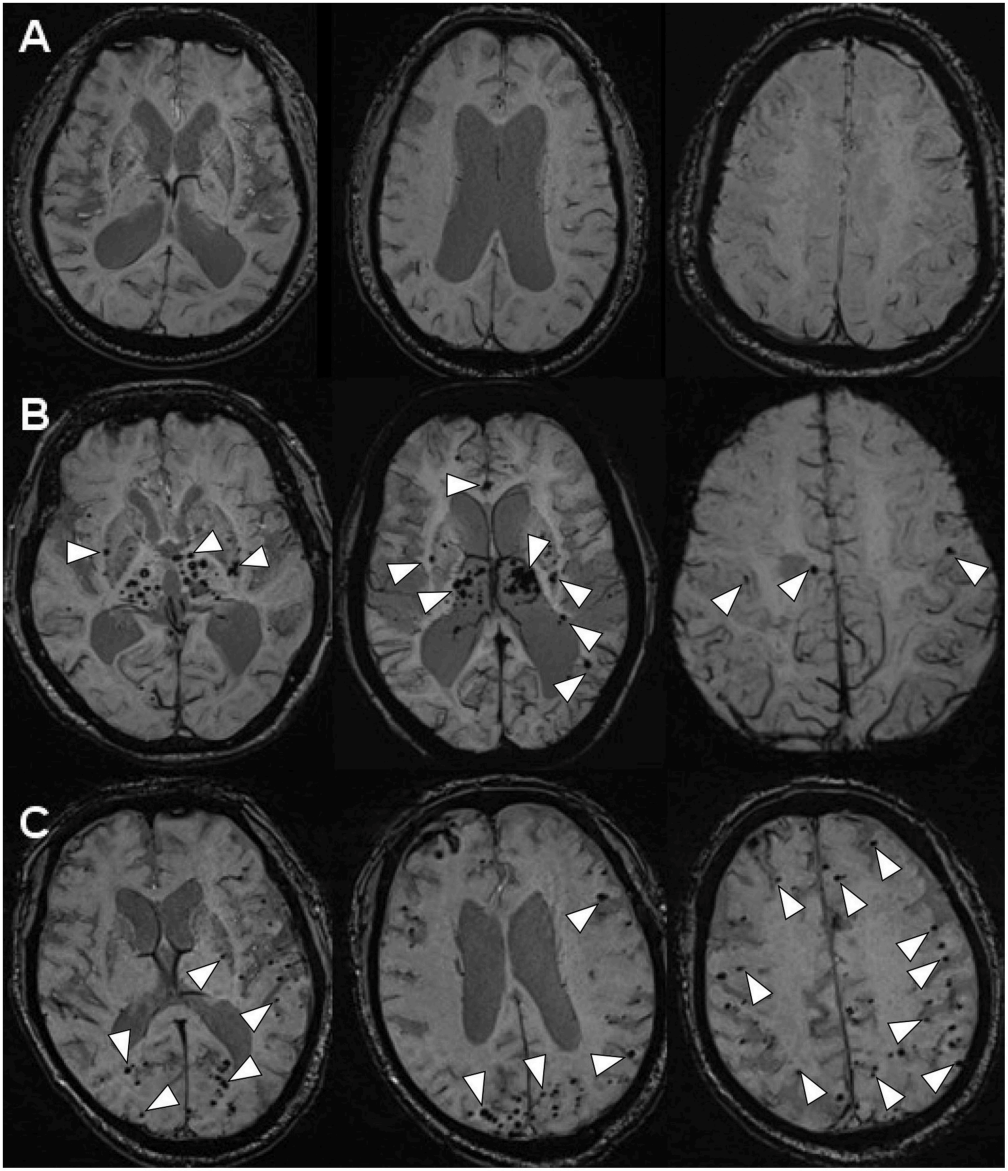
The MR-SWI neuroimaging showed three different phenomena of CMBs in the brains. **(A)** INPH: no CMBs observed in the brain (case 1). **(B)** LA: multiple CMBs distributed in deep brain structures (the basal ganglia, the thalami, the corpus callosum, the internal capsule, external/extreme capsule) (case 2). **(C)** CAA: multiple CMBs distributed in the area of cerebral lobars (the cerebral cortices and the subcortical white matter) (case 3).

The patient was a 65-year-old man who presented with memory loss and unsteadiness while walking. He had a history of hypertension for 10 years and diabetes for 3 years. He had smoked for several decades but no history of drinking. Neurological exam clearly revealed the consciousness. His mini-mental state examination (MMSE) score was 24. Strength was normal in all four extremities with exaggerated deep tendon reflexes. He had a spastic gait because of the increased muscular tension in his lower limbs. Babinski's signs on both sides were positive. The cerebrospinal fluid (CSF) pressure was 180 mmH_2_O, but with normal biochemical analysis. The conventional brain imaging demonstrated ventricular dilation (Evan index >0.3), periventricular and deep white matter changes significantly (Figure 1A). SWI showed no CMBs in the brain (Figure 2A). This patient was diagnosed as typical INPH and received V-P shunt surgery. His symptoms improved considerably after 1 month after the intervention. The Barthel index of activities of daily living (ADL) score improved from 55 to 75 and the functional activities questionnaire (FAQ) score improved from 25 to 18.

### Case 2

The patient was a 62-year-old woman who presented with gait disturbances. She had suffered from high blood pressure for 7 years. Neurological exam clearly revealed consciousness. The MMSE score was 25. She walked slowly and unstably. She was mild muscular rigidity and hyperreflexia. Babinski's signs on both sides were positive. The CSF pressure was over 160 mmH_2_O, but with normal biochemical analysis results. The conventional brain imaging also demonstrated ventricular dilation (Evan index >0.3) and significant changes in periventricular and deep white matter (Figure 1B). However, SWI showed multiple CMBs predominantly located in deep brain structures including the basal ganglia, the thalami, the corpus callosum, the internal capsule, and external/extreme capsule(Figure 2B). The surgeons thought that the V-P shunt surgery would not be suitable for this patient, who did not suffer from INPH but LA. For this reason, this patient who had hypertension only received oral tablets containing nifedipine, an antihypertensive drug. However, there was no improvement in her symptoms after drug therapy. The Barthel index of ADL and FAQ did not differ before and after treatment, showing scores of 70 and 8, respectively.

### Case 3

The patient was a 79-year-old man who presented with memory loss, gait disorder, and urinary incontinence. He had no hypertension or diabetes and no bad personal habits. Neurological examination clearly revealed the consciousness. The MMSE score was 16. He had normal strength in all four extremities with exaggerated deep tendon reflexes. He had small-stepped gait and the Babinski's signs on both sides were positive. The CSF pressure was 120 mmH_2_O below, but with normal biochemical analysis. The conventional brain imaging demonstrated ventricular dilation (Evan index >0.3), periventricular and deep white matter changes significantly (Figure 1C). SWI showed multiple CMBs distributed in the area of cerebral lobars including the cerebral cortices and the subcortical white matter (Figure 2C). Finally, this patient was not diagnosed with INPH and LA, but with CAA. No efficient interventions were recommended for CAA, and his symptoms had not improved after 1 month. The Barthel index of ADL and FAQ scores were 20 and 26, respectively. Moreover, the patient suffered from severe dementia, slight gait disturbance, and urinary incontinence, and he could not live independently.

## Discussion

All three cases met the diagnostic standard of promising INPH according to either the guidelines of international version or the Japanese second edition: (1) Individuals were older than 60 years; (2) Each had more than one of the clinical triad: gait disturbance, urinary incontinence, or cognitive impairment; (3) Brain imaging showed ventricular dilation (Evan index >0.3) and periventricular and deep white matter changes; (4) CSF pressure was <200 mmH_2_O, and the CSF content were normal; and (5) Secondary hydrocephalus had been excluded (3, 7, 8).

Scanning by T1-weighted MR, T2-weighted MR and FLAIR-MR imaging showed exactly the same clinical brain images among INPH, LA, and CAA patients (Figure 1) (Supplementary Figure 1). If we applied SWI, those three persons then varied from one another mostly in the presence and distribution of CMBs (Figure 2). Case I showed multiple vascular risk factors, but with no CMBs in the brain, as indicated by MR-SWI scanning, and he was considered suitable for V-P shunt treatment. Case II showed multiple CMBs predominantly located in deep brain structures, and these were considered to be associated with hypertensive arteriopathy (9–11). Case III also showed multiple CMBs, but they were apparently distributed in the cerebral lobars and cortical subarachnoid hemorrhage, which was supposed to be the feature of CAA (12). Although case 3 presented with cerebral atrophy in MRI, indicating that INPH occurred primarily in the elderly, it was not an exclusion criterion for definition of INPH, based upon the guidelines of Japanese second edition (3). Therefore, among these cases, only Case I underwent V-P shunt surgery. Indeed, the symptoms of that patient with typical INPH were considerably improved after the surgery. Given the information about potential outcomes of Case II and III after V-P shunt, for whom surgical intervention was not recommended, we recommend MR-SWI as a prerequisite for V-P shunt operation to thoroughly distinguish INPH from VDs and to efficiently ensure the surgical effectiveness and safety of V-P shunt operation.

Our study perspective could be supported by one previous study showing that INPH patients with cerebrovascular disease (CVD), such as LA or stroke, had poorer short- and long-term treatment outcome than those patients without CVD (13). Moreover, both studies also showed normal pressure hydrocephalus (NPH) with LA could not obtain a favorable outcome after V-P shunt surgery and they considered LA as a negative factor in decisions regarding V-P shunt treatment (14, 15), although several studies found that patients with severe LA could also benefit from the shunt surgery (16, 17). In addition, Alzheimer's disease (AD) has been shown to frequently coexist with INPH, and patients presenting with AD are less likely to obtain a fully favorable long-term outcome of shunt surgery than in those persons without AD (18). Currently, experts do not recommend against shunting in INPH patients with AD because of pathological features of AD in INPH patients, which predict a worse cognitive outcome of shunt surgery (18). Recently, Elias Johansson et al found that among 14 INPH patients, two INPH patients with extensive CMB showed serious adverse events and died within 30 days after V-P shunt (19). These evidences indicate that poor outcomes could be associated with those potential comorbidities in INPH patients (20), and it is necessary to exclude them to improve the success rate of surgery for INPH through advanced MR imaging. In order to decrease misdiagnosed events and avoid the poor outcome from shunt surgery, we therefore recommend SWI as a regular MR procedure to distinguish independent INPH without CMB from LA and CAA before V-P shunt treatment.

## Conclusion

INPH, LA, and CAA might co-exist together and also share the same clinical symptoms in elders. MR-SWI was showed to precisely distinguish independent INPH from vascular dementia including LA and CAA. Therefore, we fully recommend that SWI should be incorporated into the international identification criteria, Japanese 3rd version of INPH identification, and strongly suggested it as a routine pre-operative examination to identify independent INPH before V-P shunt treatment.

## Ethics Statement

This study was approved by Xiamen ethical committee. Written informed consent was obtained from the patients for the publication of this case report. They have consented to the submission of the case report for publication in the journal.

## Author Contributions

W-QH and H-NL contributed to collection of the study data, drafting of the manuscript, and critical revision of the article. QL and C-MT contributed to the study concept and design, image analysis, and interpretation of the data.

### Conflict of Interest Statement

The authors declare that the research was conducted in the absence of any commercial or financial relationships that could be construed as a potential conflict of interest.

## Supplementary Material

Supplementary Figure 1 The brain T1-weighted and T2-weighted MR imaging. Both T1-weighted **(A–C)** and T2-weighted MRI **(D–F)** demonstrated the similar image feature in all of three cases. **(A,D)** denoted as INPH (case 1); **(B,E)** denoted as LA (case 2); **(C,F)** denoted as CAA (case 3).

## Abbreviations

CAA: cerebral amyloid angiopathy
CMBs: cerebral microbleeds
CSF: cerebrospinal fluid
FLAIR: fluid-attenuated inversion recovery
fMRI: functional magnetic resonance imaging
INPH: idiopathic normal pressure hydrocephalus
MMSE: mini-mental state examination
MR: magnetic resonance
LA: leukoaraiosis
SWI: susceptibility weighted imaging
VD: vascular dementia
V-P: ventriculo-peritoneal.
