# Supplementary figures and images for: Susceptibility Weighted Imaging (SWI) Recommended as a Regular Magnetic Resonance Diagnosis for Vascular Dementia to Identify Independent Idiopathic Normal Pressure Hydrocephalus Before Ventriculo-Peritoneal (V-P) Shunt Treatment: A Case Study
Source: Front Neurol. 2019 Mar 29;10:262. doi: 10.3389/fneur.2019.00262 (PMC6449466; doi:10.3389/fneur.2019.00262)

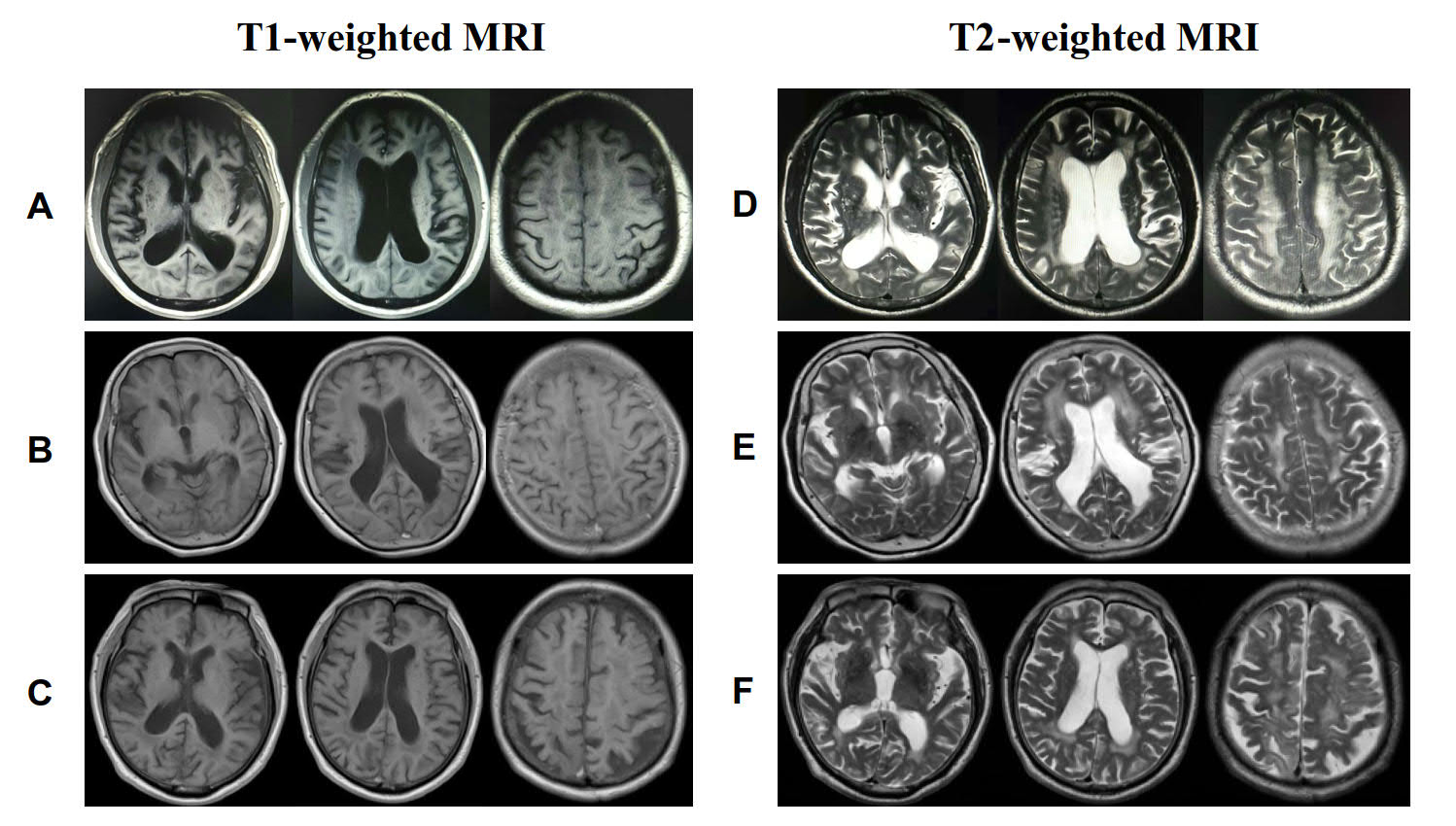

Supplement: Supplementary Figure 1 — The brain T1-weighted and T2-weighted MR imaging. Both T1-weighted (A–C) and T2-weighted MRI (D–F) demonstrated the similar image feature in all of three cases. (A,D) denoted as INPH (case 1); (B,E) denoted as LA (case 2); (C,F) denoted as CAA (case 3). [file Image_1.JPEG]
